# A core laboratory for the generation of quality-controlled g-herpesvirus bacmids: generation of KSHV microRNA mutants

**DOI:** 10.1186/1750-9378-7-S1-P27

**Published:** 2012-04-19

**Authors:** Brian Krueger, Karlie Plaisance, Rajnikumar Sangani, Curtis Lanier, Vaibhav Jain, Jianhong Hu, Rolf Renne

**Affiliations:** 1 Department of Molecular Genetics and Microbiology, University of Florida, Gainesville, FL, USA

## 

Kaposi’s sarcoma-associated herpesvirus (KSHV) encodes 12 viral microRNAs that are expressed during latency. Research into the function of these microRNAs has suffered from the lack of an experimental system that allows for the systematic removal of individual microRNAs. Here we have used the *E. coli* Red recombination system in conjunction with a new bacmid background, 219BAC, generated in the Jung Lab to create mutants for every known KSHV microRNA. The specific microRNA deletions or mutations and the integrity of the viruses has been strictly quality controlled using PCR, restriction digestion and sequencing based assays. In addition, stable viral producer cell lines for wildtype, ΔmiR-K12-1, ΔmiR-K12-3, and ΔmiR-K12-11 have been created in iSLK cells generously provided by Don Ganem. Deep sequencing was employed to sequence verify all of the current producer cell line mutants and a qPCR assay was used to verify the expression of the remaining viral microRNAs. Creation of producer cell lines for all of the microRNA mutants is ongoing and these viruses will be made available to the research community for further study.

This project was funded by ARRA RC2CA148407 to RR.

